# Supplementary material for: Are carbon emissions trading and green financial instruments synergistic? -Comprehensive quantitative research based on content analysis
Source: PLoS One. 2024 Mar 7;19(3):e0298601. doi: 10.1371/journal.pone.0298601 (PMC10919601; doi:10.1371/journal.pone.0298601)
Supplement: S1 Data — (ZIP) [file pone.0298601.s001.zip › Data/地级市Z_F工作报告.zip!/╣π╢1⁄2/╣π╓▌╩╨/└┤╫╘╣1⁄2╓┌║┼í╛┬φ┐╦╩2╛▌═°í┐_2017.docx]

各位代表：

我代表市人民政府向大会作政府工作报告，请予审议，并请政协委员和其他列席人员提出意见。

一、过去五年工作回顾

本届政府任期五年，在党中央、国务院和省委、省政府及市委的坚强领导下，全面贯彻党的十八大和十八届三中、四中、五中、六中全会精神，深入贯彻习近平总书记系列重要讲话精神和治国理政新理念新思想新战略，主动适应引领经济发展新常态，坚持稳中求进工作总基调，统筹推进稳增长、促改革、调结构、惠民生、防风险、保稳定各项工作，圆满完成了市十四届人大确定的主要目标任务。

——经济综合实力迈上新台阶。五年来，经济保持较快增长，地区生产总值由2011年的1.24万亿元预计提高到2016年的1.96万亿元，人均地区生产总值突破2万美元；地方一般公共预算收入由960亿元增加到1394亿元。发展潜力持续增强，内需对经济增长的贡献率由66.7％提高到91.1％，外贸在全国形势总体不景气的情况下保持平稳，累计实际使用外资260亿美元。广州开发区持续位居全国国家级开发区前列，南沙新区、南沙自贸试验区上升为国家战略，增城开发区加速发力，各具特色的产业功能区初具规模，总部企业增加100家，为经济持续增长打下了良好基础。

——产业结构继续优化。五年来，三次产业比重由1.6︰36.9︰61.5调整为1.2︰30.2︰68.6。服务业比重持续提高，现代服务业占比65％，金融业增加值翻一番，跨境电商进出口规模全国第一，旅游综合竞争力位列副省级城市第一，工业设计、文化创意、融资租赁等新业态发展迅速，连续3次荣登福布斯中国大陆最佳商业城市榜首。先进制造业发展稳健，完成700多家规模以上工业企业技术改造，形成5个千亿级、26个百亿级产业。战略性新兴产业持续保持两位数增长，高新技术产品产值占规模以上工业总产值比重达46％。农业保持平稳发展，农业增加值达到260亿元，比2011年增长10％以上，“米袋子”“菜篮子”安全有效供给水平持续提高。自主创新能力有较大提升，获批为国家自主创新示范区和全面创新改革试验核心区，高新技术企业由1253家发展到4700多家，科技企业孵化器面积超过800万平方米，新型研发机构44家，日均注册商标数量居副省级城市首位。

——城市综合承载力不断提升。《广州市城市总体规划（2011－2020）》获国务院批复，“三规合一”走在全国前列。空港、海港、铁路港、信息港等一批基础设施建设全面提速，2016年港口货物吞吐量5.4亿吨，集装箱吞吐量1875万标箱，分别位居全球第6、第7位；机场旅客吞吐量5968万人次，货邮吞吐量163.8万吨，均位居国内主要机场前列；贵广、南广高铁等4条铁路相继开通，高速公路通车里程972公里，地铁通车10条（段）、308公里。入选国家综合运输服务示范城市、地下综合管廊试点城市。城市更新有序推进，“幸福同德围”“美丽金沙洲”和广钢新城、罗冲围建设取得成效，48个老旧小区微改造项目稳步实施，琶洲互联网创新集聚区、国际金融城、万博城等一批新的发展载体建设全面铺开。新农村建设明显加快，农村集体“三资”交易管理平台全面建成，完成89条市级美丽乡村试点建设和6个镇、116条村的名镇名村创建任务。

——城市环境逐步改善。五年来，城市空气治理持续推进，PM2.5等主要污染物平均浓度持续下降。新增污水处理能力34万吨/日，建成污水管网692公里，城市集中式饮用水水源地水质达标率保持100％，完成168.6万农村人口自来水改造。垃圾处理设施建设取得突破，垃圾分类处理荣获联合国“中国城市可持续发展范例奖”。城市绿化取得新进展，全市森林覆盖率42.14％，建成绿道3200公里、碳汇造林21万亩，新增森林公园33个、湿地公园19个，建成区绿化覆盖率41.8％，荣获全国国土绿化突出贡献奖。城市秩序持续好转，下大力气整治违法建设、违法施工、违法户外广告和“六乱”，城中村综合整治和专业市场安全隐患整治取得成效。平安广州建设成效明显，社会治安综合治理深入推进，警情下降、立案下降、破案率上升、群众满意度上升。成功创建全国质量强市示范城市，“食得放心”城市建设全面推进。完成2.25万来穗人员积分入户。安全生产形势持续稳定，安全生产责任体系、应急管理体系和防灾减灾救灾体系不断健全，城市环境干净整洁平安有序。

——改革开放取得新突破。五年来，完成新一轮政府机构改革和行政区划调整，事业单位分类改革稳步进行。“放管服”改革取得实效，取消、下放行政审批和备案事项285项。金融和财税改革扎实开展，在全国率先建成民间金融街，6个金融功能区、5个金融交易平台加快发展，上市公司累计达133家，新三板挂牌企业累计达348家；全面推开营改增试点，政府性债务余额下降14％，财政透明度位居全国前列。国企改革不断深化，实现国有资产统一监管，近七成市属国企完成混合所有制改造，国有企业资产总额、利润总额年均保持两位数增长。南沙自贸试验区累计形成209项改革成果，85项经验在全国、全省复制推广。国际友好城市和友好合作交流城市增至65个，驻穗总领事馆55个，连续4届担任世界城市和地方政府组织联合主席城市，城市影响力明显增强。

——人民生活水平进一步提高。五年来，坚持每年办好十件民生实事，民生和社会事业支出占财政支出总额的76％。累计新增城镇就业146万人，社保缴费3016万人次，企业退休人员、农转居、城乡居民养老金持续提高，城乡低保标准分别提高80％和123％、实现并轨，新开工和筹集保障性安居工程住房11.29万套。学前教育公益性普惠性投入加大，职业教育、高等教育办学实力明显增强，普通高考一本上线率比2011年提高9.7个百分点。实施分级诊疗、双向转诊，建成一批基层医疗服务设施，基层医疗卫生机构综合改革取得显著成效，有效应对登革热、禽流感、埃博拉等重大传染病疫情，居民平均期望寿命81.72岁。广州图书馆新馆、广州少儿图书馆新馆、粤剧艺术博物馆、13个儿童公园建成开放，推进广州文化馆、南汉二陵博物馆、职业技术院校迁建工程等一批文化、教育、卫生、民政设施建设，《广州大典》一期编纂出版，话剧《共产党宣言》获文华奖，成功创建全国版权示范城市。在全国率先完成地方足协和足球改革，举办世界羽毛球锦标赛、广州马拉松赛、广州国际龙舟邀请赛、亚冠联赛等重大体育赛事，竞技体育获6项奥运冠军，全民健身和体育产业取得新成绩。实现全国双拥模范城八连冠，荣获全国少数民族流动人口服务管理示范城市称号。

——政府自身建设得到加强。五年来，依法向人大及其常委会报告工作，向人民政协通报情况。累计办理市人大代表建议1925件、政协提案2548件。提请市人大常委会审议地方性法规草案23件，制定修改政府规章172件。认真听取民主党派、工商联、无党派人士及工会、共青团、妇联等人民团体意见。开展法治政府示范区和依法行政示范单位创建活动，建立重大行政决策和政府合同合法性审查机制，推行政府法律顾问制度，严格行政执法监督，法治政府建设走在全国前列。实行重大民生决策公众咨询监督制度，多种形式发布政府常务会议信息。建立舆情反映事项和处理机制。创新“五个一”基层治理模式，市、区、街（镇）、村（居）四级政务服务体系全覆盖，整合41个部门和各区的74条热线，统一设立12345政府服务热线，“一窗办事”“一号受理”“一网服务”在全国推广，阳光政府建设有新突破。严格落实中央八项规定精神，“四风”问题得到有效遏制；大力推进领导干部履行经济责任审计，权力运行监督进一步强化，廉洁风险防控进一步加强，一批腐败分子得到惩处，廉洁政府建设取得新成效。

过去五年的发展成就，集中展现了全市人民的无穷创造力，极大增强了建设国家重要中心城市的自信心和凝聚力，必将激励全市人民在实现“两个一百年”奋斗目标的新征程上奋力前行。

各位代表，2016年是本届政府收官之年。我们在以习近平同志为核心的党中央坚强领导下，坚持创新、协调、绿色、开放、共享的发展理念，坚持以人民为中心的发展思想，坚持以提升综合实力和可持续发展能力为目标，以推进供给侧结构性改革为主线，攻坚克难，主动作为，经济社会发展稳中向好。地区生产总值预计增长8.1％，地方一般公共预算收入可比增长5.5％，居民消费价格指数上涨2.7％，城镇登记失业率2.4％。主要做法和特点是：

（一）积极应对经济下行压力，经济保持平稳增长。我们贯彻国家和省的决策部署，采取一系列措施，稳定经济增长。

一是投资更加有效。制定创新重点领域投融资机制鼓励社会投资实施意见，出台创新完善中小微企业投融资机制十条措施，促进投资增长。创新投融资方式，推出一批政府和社会资本合作（PPP）项目，成立广州铁路投资建设公司。推进重点项目建设，安排186个重点项目、完成投资1256亿元，完成年度投资计划的119％，全市固定资产投资5704亿元，增长8％。

二是消费加快升级。开展国内贸易流通体制改革，大力培育拓展电子商务、信息消费、旅游休闲等消费新增长点，优化城市核心商圈功能，推动商贸转型升级。实现社会消费品零售总额8706亿元，增长9％。

三是外贸平稳向好。出台促进进出口稳定增长若干意见，推行出口退税网上申报和限时办结等制度，全面促进通关便利化。推进服务贸易和服务外包发展，开展飞机租赁、汽车平行进口等业务，组织市场采购贸易试点，发展跨境电商、保税贸易等外贸新业态。实现商品进出口总值8567亿元，增长3.1％，其中出口5187亿元，增长3％。

（二）实施创新驱动发展战略，城市发展动力日益增强。我们坚持把创新驱动发展作为核心战略和总抓手，全力推进国家创新中心城市和国际科技创新枢纽建设。

一是创新主体蓬勃发展。大力实施财政科技经费倍增计划，市、区两级财政投入81.3亿元。实施科技创新小巨人企业和高新技术企业培育行动，对2307家企业进行研发投入后补助，新增高新技术企业2800多家，科技创新企业超过12万家。安排10亿元工业转型升级专项资金，扶持115个工业企业技术改造投资项目。

二是创新载体不断壮大。推进国家自主创新示范区建设，加快科技创新走廊发展。科学城获批全国首批区域双创示范基地，国家商标品牌创新创业（广州）基地、国家工商总局商标审查协作广州中心落地运行，建成众创空间99家，国家级优秀孵化器数量居全国前列。拓展中以、中瑞、中欧等国际创新合作，国际科技合作基地建设顺利推进。

三是创新生态逐步优化。落实“1＋9”科技创新政策，设立科技型中小企业信贷风险补偿资金池，建立中小企业发展基金和知识产权质押融资风险补偿基金，成立中小微企业融资再担保公司，风险投资、创业投资机构达1500家。出台集聚现代产业人才“1＋4”文件，发出人才绿卡1180张，新入选国家“千人计划”专家46人、“万人计划”专家52人。举办2016“创响中国”巡回接力首站活动，中国创新创业成果交易会、“小蛮腰”科技大会落户广州，参加中国创新创业大赛的企业和团队达2054家。加强知识产权保护，行政处理知识产权类案件2576件，结案率99.3％，发明专利申请量增长58.8％，PCT国际专利申请量增长163.6％，增速均位居全国前列。

（三）推进供给侧结构性改革，产业结构持续升级。我们认真落实中央、省关于供给侧结构性改革的决策部署，出台“1＋5”政策文件，着力在补短板、降成本上下功夫，提升供给体系质量和效率。

一是创新型经济加快成长。出台实施广州制造2025、加快发展生产性服务业三年行动方案等政策，推进服务贸易创新发展、跨境电商综合试验区等试点，培育经济增长新动能。新产业快速成长，先进制造业增加值增长7.5％，LG8.5代液晶面板项目二期、广汽乘用车第二生产线、广汽菲克工厂投产，北汽威旺下线，新能源汽车产量增长1倍。中远海运散货、思科中国创新中心、GE生物产业园、富士康10.5代显示器全生态产业园等一批总部企业落户。新业态持续壮大，网上商店零售额增长20.7％，旅游购物商品出口增长18.1％，保税物流进出口增长24.5％，旅游业总收入增长12％，全国“互联网＋”十大标杆城市位居第三。

二是降成本取得新进展。深化“放管服”改革，出台新版行政审批备案事项目录，精简行政审批许可备案事项176项。全面启动“一窗式”集成服务，放宽商事主体登记条件，实施工商登记全城通办。全面推开营改增，减免52项行政事业性收费、8项政府性基金，落实降低四险一金缴费费率政策。全年为企业减负超过700亿元，规模以上工业企业每百元主营业务收入成本比全国、全省平均水平分别低2.9元和1.9元。

三是“三去”工作扎实有效。用市场化法治化手段，完成410户国有“僵尸企业”出清重组，淘汰印染行业落后产能7846万米。出台促进房地产市场平稳健康发展意见，保持房地产政策的稳定性和连续性，一手住宅去库存周期降至6.3个月，一手商服物业去库存周期降至14.6个月。开展互联网金融风险专项整顿，严厉打击非法集资行为，银行机构不良贷款率1.33％，比全国低0.48个百分点，企业直接融资规模和增速居全国前列，降低企业融资成本超过200亿元。

（四）加强城市规划建设管理，城市治理水平明显提升。我们认真贯彻中央和省城市工作会议精神，出台加强城市规划建设管理工作实施意见，加快建设枢纽型网络城市。

一是三大国际战略枢纽建设成效显现。推进国际航运枢纽21个工程项目，南沙港三期建成投产，建设无水港及办事处24个，新增8个国际友好港和12条国际班轮航线，“珠江航运运价指数”上线，邮轮旅客规模跃居全国第三。建设国际航空枢纽8个工程项目，第二航站楼主体结构完成，国家级临空经济示范区获得批复，东方航空广东分公司落户，率先推动口岸查验配套服务改革，新科宇航第二飞机维修机库竣工投产，新开通、复航及加密国际航线27条，国际旅客吞吐量增长19.5％。国际科技创新枢纽24个工程项目完成投资229亿元，琶洲互联网创新集聚区9个项目开工。

二是综合交通枢纽建设取得新突破。广州国际性综合交通枢纽建设纳入国家“十三五”规划，推动国家、省、市共建综合交通枢纽示范城市，完成广州铁路枢纽总图规划。3个国铁项目、6个城际轨道项目、8个高速公路项目、11条地铁项目建设进入主体施工阶段，广清高速扩建工程、广州大桥新桥和地铁六号线二期、七号线一期建成通车。

三是城市环境持续改善。全面完成燃煤电厂机组超洁净排放改造，整治挥发性有机物和道路扬尘污染、船舶污染，空气质量持续向好，PM2.5平均浓度36微克/立方米，下降7.7％。深入实施“河长制”，组织河长培训，建立问责机制。16条广佛跨界河涌整治工程完成率85％，列入国家监管的35条河涌已有6条消除黑臭，新改（扩）建8座污水处理厂，建成污水管网300公里。5座资源热力电厂全面开工，2座资源热力电厂点火运营，新增管道燃气用户58万户。完善城市更新政策，推进42个旧村、284个旧厂、20个旧城项目更新改造。建设绿色生态网络，成功创建全国绿化模范城市。

（五）积极扩大对外开放，城市国际化水平有新提升。我们充分发挥对外开放先发优势，提高参与全球城市竞争与合作的层次。

一是参与“一带一路”建设取得成效。推动企业走出去，在境外设立企业（机构）260个。举办21世纪海上丝绸之路国际博览会主题论坛，推进海上丝绸之路申遗工作。开通中欧班列，加强沿线城市产能合作项目建设，赴沿线国家和地区投资额增长1.45倍，吸收合同外资增长1.63倍，与沿线国家和地区进出口占全市进出口总额的25％。

二是国际合作更加密切。举办广州国际投资年会、夏季达沃斯“广州之夜”、世界经济论坛商业圆桌会议、亚欧互联互通媒体对话会等国际会议。开展国际城市创新“一奖一会一节”和广交会、广博会、海交会、金交会、国际旅游展等国际交流活动。成功申办2017年《财富》全球论坛、2018年世界航线发展大会，国际金融论坛全球年会永久落户广州。

三是南沙自贸试验区加快建设。出台支持南沙自贸试验区建设的若干意见。加大制度创新力度，全年形成100多项改革创新成果，31项在全省复制推广，市场监管企业信息信用平台等政策走在全国前列。推进“三互”大通关改革，国际贸易“单一窗口”升级版上线运行。重点组团建设有序推进，“一核四区”功能布局主骨架初步形成。新设企业1.4万家，新增金融及类金融企业820家，中交、中铁、中铁建等功能型总部落户，广汽丰田三期等骨干项目开工。

（六）切实保障和改善民生，社会事业全面进步。我们坚持以民之所望为施政所向，保基本、兜底线、建机制，让发展成果惠及人民群众。2016年十件民生实事全部完成，兑现了政府承诺。

一是就业和社保水平稳步提高。把就业作为民生的头等大事，建设253个创业基地，新增就业31万人，城乡居民人均可支配收入分别增长8.5％和10％。出台公共租赁住房保障办法，新开工和筹集保障性安居工程住房2.8万套（户）。建立事实无人抚养儿童基本生活保障制度，为4.8万名低保对象发放救助金4．1亿元，城乡低保标准提高至840元/月，医疗救助最高标准每人每年提高到35万元。推进全国养老服务业综合改革试点，每千名老人养老床位40张。

二是教育、文化、卫生等事业取得积极进展。促进义务教育均衡优质发展，实现全国义务教育发展基本均衡区全覆盖，完善来穗人员随迁子女接受义务教育政策，启动129所中小学新改扩建工作、新增2万个学位，出台2所高水平大学建设方案，2所高职院校纳入省一流高职院校建设。发展文化事业，举办羊城国际粤剧节、央视春晚广州分会场等大型活动，开展系列文化惠民活动。推进住院医师规范化培训，建立儿科医疗专项补助机制，保障全面两孩政策实施。推进体育惠民，303家体育场馆优惠或免费开放，新建社区小型足球场26个，首创全民健身公共服务平台。

三是社会治理创新力度加大。创新社会治理体制，超过55％的社区建成幸福社区。建成10个区级、162个镇（街）级社区网格化服务管理中心，网格事件办结率96％以上。推广“民主协商、一事一议”基层治理经验，一村（社区）一法律顾问全面落实，采取社区、社会组织、专业社工“三社联动”服务社区居民。有效化解一大批信访积案。开展社会组织公益创投活动，“政府主导、社会协同、项目运作、专业服务”的社工发展模式在全国推广。组织来穗人员融合社区试点，完善立体化、信息化社会治安防控体系。开展“食品安全年”活动，创建国家食品安全城市。组建3315人专职安监员队伍，开展安全生产专项整治和打非治违，有效防御“妮妲”等台风，全年未发生重特大安全事故和群体性职业病危害事件。开展“羊城慈善为民”行动，深化扶贫济困工作。积极开展援疆、援藏等对口支援帮扶，广清结对帮扶获全省对口帮扶第一名，顺利完成市内农村扶贫任务。

其他各项社会事业全面发展。宗教团体和场所规范化管理水平提升。普法和法律服务、社区矫正、人民调解、监狱和戒毒管理工作进一步加强。连续3次获评全国人民防空先进城市。国防动员、海防打私、打假、妇女儿童、残疾人、侨务、对台、贸促、口岸、应急、气象、地震、统计、参事、文史、档案、保密、供销、修志、仲裁、红十字会等工作取得新进步。联合国开发计划署发布人类发展指数广州蝉联全国第一。

各位代表，过去五年的成绩来之不易，这是党中央、国务院和省委、省政府及市委坚强领导的结果，是市人大、市政协大力支持、有效监督的结果，是全市人民团结奋斗的结果。我代表广州市人民政府，向全市人民、驻穗部队官兵、各民主党派、人民团体以及社会各界人士表示崇高的敬意！向关心支持广州改革发展的港澳台同胞、海外侨胞、国际友人表示衷心的感谢！

在肯定成绩的同时，我们也清醒看到，经济社会发展还存在一些问题：一是经济增长质量效益有待提高，内生动力不足，先进制造业新增长点不多，工业投资不足，科技创新贡献率不高，现代服务业还有待进一步加强，重大骨干项目较少，财政收支压力大。二是城市治理精细化品质化水平有待提升，大气、水、土壤、垃圾等污染治理任务繁重，河涌水环境共管共治机制建设有待加强，城市交通拥堵问题没有得到缓解，部分老旧小区、城中村环境问题比较突出。三是营商环境需要进一步优化，国企改革有待深化，民间投资进入领域有待放宽，个别改革事项制约因素多、推进比较慢。四是城乡和区域间基本公共服务均等化水平有待增强，优质教育、医疗资源分布不均衡，人民对食品安全、公共安全改善要求迫切，部分农村地区环境问题突出，民生事业发展与群众期望还有差距。五是政府职能转变不够到位，行政办事效率仍需提高，不想为、不敢为的现象仍然存在，一些领域腐败案件时有发生。对此，我们必须高度重视，勇于担当，全力加以解决。

二、今后五年的目标任务

未来五年，是落实新发展理念的关键五年。今后五年政府工作总体要求是：高举中国特色社会主义伟大旗帜，坚持以马克思列宁主义、毛泽东思想、邓小平理论、“三个代表”重要思想、科学发展观为指导，深入贯彻习近平总书记系列重要讲话精神和治国理政新理念新思想新战略，落实中央、省委和市委决策部署，统筹推进“五位一体”总体布局和协调推进“四个全面”战略布局，坚持稳中求进工作总基调，牢固树立和贯彻落实新发展理念，适应把握引领经济发展新常态，以提高发展质量和效益为中心，以推进供给侧结构性改革为主线，大力实施创新驱动发展战略，高水平建设国际航运中心、物流中心、贸易中心、现代金融服务体系和国家创新中心城市，强化枢纽型网络城市功能，提高全球资源配置能力，推动国家重要中心城市建设全面上水平，努力让全市人民生活更加幸福美满。

今后五年经济社会发展主要目标是：经济发展质量效益显著提高，枢纽型网络城市格局基本形成，人民生活品质大幅提升，全面完成“十三五”发展目标任务，为率先基本实现社会主义现代化打下坚实基础。2018年率先全面建成小康社会，2020年地区生产总值达到2.8万亿元，年均增长7.5％以上。实现上述目标，我们着力在以下几个方面狠抓落实：

——推动全面深化改革走在前列。围绕建设市场化国际化法治化营商环境，加快政府职能转变，深化“放管服”改革，充分发挥市场在资源配置中的决定性作用和更好发挥政府作用。围绕推进经济体制改革，重点抓好财税、金融、国企、投融资和农村等领域的改革试点。围绕加大公共服务领域改革力度，深化教育、医疗卫生、养老、安全生产等综合改革。围绕完善财政事权与支出责任相匹配的发展体制，推进市、区财政和城市管理体制改革，调动各区发展积极性。

——提高经济发展质量效益。坚定不移推动产业转型升级，构建高端高质高新产业新体系，全面实施制造强市战略，培育具有核心竞争力的企业主体，着力振兴实体经济。全面提高全要素生产率，降低单位地区生产总值能耗。到2020年，服务业增加值占地区生产总值比重70％，现代服务业增加值占服务业增加值比重70％，先进制造业增加值占规模以上制造业增加值比重70％。

——推动国家创新中心城市建设。建设国家自主创新示范区和全面创新改革试验核心区，推动形成以创新为引领和支撑的经济体系和发展模式。大力培育高新技术企业和创新型企业，加大创新投入，保护知识产权，推进科技与金融、产业融合发展。到2020年，高新技术企业8000家，科技创新企业20万家，广州国家自主创新示范区以国际先进科技园区为标杆，实现并跑国家一流示范区。建设领军人才集聚地，打造海内外人才发展平台，实施更加开放的人才政策，保护和激发企业家创业家精神，建设人才高地。

——实现新一轮高水平开放合作。深化“一带一路”战略枢纽城市建设，加大引资引技引智力度，集聚全球科技、人才、资本等资源。实施优进优出战略，建设国际采购、配送中心，不断强化国际供应链体系。拓展城市外交合作平台，开放合作融入全球创新网络。推动重点功能组团建设，加大产业导入力度，加快自贸试验区制度创新，建设南沙城市副中心，把南沙自贸试验区打造成高水平对外开放的门户枢纽。

——建设枢纽型网络城市。完善城市功能和生产力布局，实施“枢纽＋”战略。建设国际航运、航空、科技创新枢纽。打造精品珠江，优化提升一江两岸三带。强化中心城区与外围城区之间互联互通，完善大交通综合网络体系，形成全市30分钟交通圈，建设国际性综合交通枢纽。加快信息基础设施建设，实施“互联网＋”行动和大数据战略，建设国家新型智慧城市先行区。

——统筹城乡区域发展。加强城市发展战略研究，推动“多规合一”，持续系统推进差异化城市更新，提高城乡规划建设品质。补齐城乡基础设施和基本公共服务短板，健全城市公共安全体系，建设干净整洁平安有序城市环境。全面加强“三农”工作，大力发展都市现代农业。促进广佛肇清云韶融合发展，加强粤港澳和泛珠三角合作，做好对口支援帮扶，促进区域联动发展。

——推进全面依法行政。深入推进法治政府建设，率先基本建成法治政府。培育法治思维，运用法治方式，深化行政执法体制改革，全面依法行政，促进审批法治化环境建设，加大执法监督检查力度。

——提升城市文化软实力。建设国家历史文化名城，弘扬社会主义核心价值观，巩固全国文明城市成果。深化文化体制改革，加快文化产业转型升级，倡导全民阅读和家庭文明。实施文化品牌战略，加强传统文化保护利用，加大城市推介宣传力度，提升文化影响力。提高市民文明素质，弘扬“务实、求真、包容、开放、创新”的广州城市精神。

——切实增进民生福祉。聚焦就业、社保、住房等重点民生社会事业，推进卫生强市建设，打造健康广州。优化学校、医院、养老服务机构等资源布局，促进基本公共服务均等化。提高城市管理水平，完善社会治理体制机制，加强社区网格化服务管理，实施品牌社会组织战略，加强和创新社会治理，建设更高水平的平安广州。

——持续加强生态文明建设。坚持把生态文明建设放在突出位置，划定生态保护红线和控制线，推动东部生态廊道和北部生态屏障建设，加快建立和完善生态补偿机制，构建生态网络体系。开展能源和水资源消耗、建设用地等总量和强度双控行动，促进资源集约高效利用，推动绿色低碳循环发展。深入实施大气、水、土壤污染治理和垃圾治理，系统推进环境综合治理。让市民的生活、工作环境更优美，让优美环境成为广州的最大竞争力和软实力。

未来五年，我们充满期待，充满信心；未来五年，我们更要干在实处，走在前列。我们坚信，广州将率先实现全面建成小康社会目标，率先基本实现社会主义现代化的基础也将更加坚实，人民生活一定会更加幸福美好。

三、2017年工作安排

今年，是新一届政府的开局之年，是供给侧结构性改革的深化之年，我们要以优异成绩迎接党的十九大胜利召开，做好政府工作至关重要。经济社会发展主要预期目标是：地区生产总值增长8％左右，地方一般公共预算收入可比增长7.8％，固定资产投资增长12％，社会消费品零售总额增长8％左右，商品出口总值增长2％，城乡居民收入增长与经济增长基本同步，城市居民消费价格涨幅控制在3％，城镇登记失业率控制在3.5％以内，节能减排降碳约束性指标完成年度任务。

实现上述目标，我们重点抓好以下工作：

（一）加快推进创新驱动发展。坚持把创新作为第一动力，完善区域创新体系，增创自主创新优势，在珠三角国家自主创新示范区中发挥引领作用。

培育发展科技创新企业。跟踪服务4000家后备高新技术企业，培育引进一批科技创新企业，力争新增高新技术企业超过1000家、科技创新小巨人企业1500家。支持一批科技创新中小微企业、小巨人企业和创新标杆企业做大做强。支持规模以上工业企业设立研发中心，加大技术改造力度，提升产品竞争力。推动实现高新技术产品产值占规模以上工业总产值比重、高新技术制造业增加值占规模以上制造业增加值比重双提升，加快新旧动能转换。

建设重大创新平台。加快国家自主创新示范区建设，调整示范区区域范围，把示范区打造成为创新企业集聚发展高地。提升孵化器和众创空间的服务能力和专业化水平，打造“广州标准”。积极合作共建新型研发机构，支持中科院广州生物医药与健康研究院、清华珠三角研究院等新型研发机构建设，积极争取国家实验室、国家重大科技基础设施和广东实验室落户广州。建设产城融合的科技小镇。布局未来产业，加强颠覆性、关键性技术研发应用和产业化。加强开放合作，促进集成创新，推动高精尖大科技产业发展。

落实创新政策措施。推动高校和科研机构科技成果使用权、处置权、收益权改革，发展各类科技成果转化机构。推动科技、金融深度融合。落实企业研发费用税前加计扣除政策。实施加快集聚产业领军人才“1＋4”政策，用好“人才绿卡”制度，多渠道筹集供应人才和新就业职工公寓1万套，力争广州地区新增“千人计划”“万人计划”专家各70名。建设知识产权服务业集聚区，开展国家知识产权质押融资示范和重点产业知识产权运营试点。

优化创新创业环境。大幅精简科技管理流程，完善科技创新资源配置方式，营造鼓励创新、宽容失败的良好氛围。推动科技创新企业挂牌、上市。办好中国风险投资论坛，建设风投创投中心。加强与世界一流高校和科研院所的交流合作。大力开展大众创业、万众创新，继续办好中国创新创业大赛、中国创新创业成果交易会、海外人才交流会、“小蛮腰”科技大会。实施标准化战略。

（二）深化供给侧结构性改革。把满足需求作为最终目的，把提高供给质量作为主攻方向，把深化改革作为根本途径，推动经济平稳增长、稳中有进。

推进“去降补”有实质性进展。推动企业兼并重组和市场出清，整治“三无”经营行为。合理增加住宅用地供应，盘活城市闲置和低效用地，发展住房租赁市场，完善多层次住房供应体系，加大调控力度，合理引导住房消费，保持房地产市场平稳健康发展。扩大直接融资规模，降低企业杠杆率，有效防范政府债务和金融风险。继续开展降本增效行动，着力降低企业要素成本和制度性交易成本。推进创新型经济、城市建设、公共服务等领域补短板重点工程，着力补齐制度短板。

加快推进基础性关键性改革。深入推进“放管服”改革，再下放一批行政审批备案事项，规范权责清单管理。深化商事制度改革，推行工商登记全程电子化和企业名称自主申报，完善市场监管体系和社会信用体系。推动投融资体制改革，对企业投资项目实行准入负面清单、行政审批清单、政府监管清单管理。深化国资国企改革，完善现代企业制度，推动上市公司“二次混改”，开展职业经理人试点，稳妥推进员工持股计划，理直气壮做强做优做大国有企业。深化财税体制改革，出台新一轮市、区财政管理体制方案，支持推动国税、地税征管体制改革。加大教育、医疗、养老、安全生产等公共服务领域改革力度。

深入推进农业供给侧结构性改革。落实发展都市现代农业三年行动计划，加快建设广州国际种业中心，扶持发展粮食、蔬菜规模生产基地、水产健康养殖示范场、畜禽标准化示范场，建设现代化屠宰场和定点生猪供应基地。稳步推进农村集体产权制度改革。支持农业规模化生产，鼓励农民专业合作社、家庭农场、种养大户发展。推动农业机械化、信息化。健全农产品质量安全监管体系。划定永久基本农田，建设高标准基本农田。抓好农村电商、休闲农业、乡村旅游等新业态。推进海洋高技术产业基地、科技兴海产业示范基地建设。

进一步释放需求潜力。扩大有效投资，全年安排市重点建设项目345个，其中正式项目260个、投资1294亿元，带动全社会固定资产投资6390亿元。完善重点项目协调推进机制，制定重点项目供地指标、建设资金、配套设施等保障政策。落实重点领域鼓励社会投资实施意见，推出一批政府和社会资本合作试点项目。多渠道刺激消费，举办国际购物节、国际美食节等主题展贸展销活动，深化内贸流通体制改革，开展传统零售体验消费，实施电子商务示范工程，推广专业市场转型升级公共服务平台。

（三）着力振兴实体经济。坚持市场导向，强化对实体经济的支持，厚植传统优势，发展壮大电气机械、医药制造等新的支柱产业，形成四梁八柱更加稳固的产业支撑体系，提高整体竞争力。

大力发展先进制造业。实施制造强市战略，开展先进制造业创新发展四大行动。优化产业规划布局，建设黄埔、南沙、增城等7个先进制造业基地，打造5个国际汽车零部件基地、互联网产业集聚区“一核五基十镇”和集成电路产业生态圈，构建智能装备和机器人、节能和新能源汽车、生物医药与健康等先进制造业产业集群。加快中药产业发展，推进仿制药一致性评价工作。大力发展物联网、云计算、大数据、人工智能等新技术，推动新一代信息技术与制造业融合创新发展。加大吸引外资力度，推动一批世界级创新龙头项目落户，更好发挥外资企业对促进实体经济发展的重要作用，努力使广州成为全球企业投资首选地和最佳发展地。

推动服务业高端发展。出台服务经济发展规划，重点发展商务、会展、科技、信息、物流等生产性服务业，着力发展文化、旅游、体育、健康等生活性服务业。强化金融服务实体经济功能，争取设立国家绿色金融示范区，推动设立一批金融机构和金融平台，加快基金小镇、财富小镇、绿色金融小镇等特色金融功能区建设。实施促进企业集群发展办法，大力培育和引进总部企业。深化国家旅游综合改革试点，建设国际旅游目的地和集散地，创建国家文化产业示范区，促进文商旅融合发展。

培育具有核心竞争力的企业主体。支持企业间战略合作和跨行业、跨区域兼并重组，形成一批枢纽型骨干企业。加大对中小微企业扶持力度，培育一批高水平中小企业。重视优化产业布局，推动企业向园区集中。坚定不移发展民营经济，全面落实促进民营经济发展政策措施，提振民营企业家信心。培育一批高成长性民营企业，建立重点民营企业改制培育名录，建设10个民营企业（中小企业）创新产业化示范基地。加快推进中国（广州）中小企业先进制造业中外合作区建设，支持企业面向全球集聚人才、技术、资金等创新资源。

营造实体经济良好发展环境。实施质量强市战略，开展重点产品质量提升行动，提高质量标准，加强全面质量管理。发扬“工匠精神”，加强品牌建设，培育更多“百年老店”。强化企业服务，着力解决制约企业发展的人才、用地、融资等瓶颈问题。依法保护企业家财产权和创新收益，满腔热情支持企业家专心创新创业，关心、尊重、爱护和服务企业发展。

（四）加大对外开放力度。顺应经济转型升级迫切需要，提高对外开放水平，在开放中激发市场活力和社会创造力。

保持外贸稳定增长。落实各项稳定外贸发展政策，组织海外市场开拓系列活动。开展市场采购、服务贸易创新等试点，建设国家跨境电商综合试验区，认定支持一批外贸综合服务试点企业。扩大汽车整车、关键零部件进口。优化整合老工业园区，推进一般贸易和加工贸易转型升级，重点发展飞机和船舶租赁、保税贸易、跨境电商等新业态。推动“三互”大通关改革和国际贸易“单一窗口”、数字口岸建设，提高口岸综合服务能力与水平。

推动高水平国际合作。加快企业走出去步伐，支持企业到“一带一路”沿线国家和地区投资兴业。参与马来西亚马六甲工业园和沙特吉赞经济城等境外园区建设，加强与南太平洋岛国和非洲国家经贸交流合作。举全市之力办好2017《财富》全球论坛，扩大城市国际影响力。加快建设侨梦苑。举办2017广州国际创新节、第25届广博会、第25届广州国际旅游展和第6届金交会，办好广州国际投资年会、21世纪海上丝绸之路博览会等高端招商活动，组织广州—奥克兰—洛杉矶三城经济联盟广州峰会、国际金融论坛全球年会和世界城市日活动，打造国际交往中心。

加强区域合作交流。推进粤港澳深度合作区开发建设，实现穗港澳服务贸易自由化。强化与泛珠三角城市和高铁沿线城市合作，加快基础设施互联互通。推进更高层次的广佛同城化、广清一体化，加快广佛肇清云韶经济圈建设，重点推进交通设施、产业园区、环境整治、公共服务等重大合作项目。坚持精准扶贫、精准脱贫，巩固市内农村扶贫成果，做好对口帮扶梅州、清远产业共建和援疆援藏援黔等工作。

（五）推进枢纽型网络城市建设。按照“枢纽＋”战略，聚焦“三中心一体系”，建设与全球资源配置能力相匹配的重大基础设施。

大力推进三大国际战略枢纽建设。推进国际航运枢纽建设，加快广州港深水航道拓宽工程、南沙港区四期、国际通用码头、南沙国际邮轮码头、疏港铁路等重点项目建设，完善多式联运体系，新增国际航线10条；发展港口物流、航运金融、船舶交易等航运服务产业。推进国际航空枢纽建设，抓紧白云机场扩建工程、白云机场商务航空服务基地等重点项目建设，加快机场第二高速等11项道路工程进度，抓好白云机场第四、五跑道、噪音安置区建设及第三航站楼前期工作，开展第二机场规划选址，新增国际航线10条以上；推动临空经济示范区建设，发展航空物流、航空维修、航空制造、航空金融、客改货等临空产业。推进国际科技创新枢纽建设，加快全面创新改革试验核心区建设，抓好科技创新走廊及珠江沿岸创新带的产业导入，推动院士专家创新创业园、软件产业集中孵化中心三期等重点项目建设，力争高新技术产品产值占规模以上工业总产值比重达47％以上，每万人发明专利拥有量达24件以上。

优化提升一江两岸三带。全面落实一江两岸三带核心段建设方案，加快建设30公里精品珠江。推动游船码头、堤岸护栏等重点改造工程，完成建筑立面整饰、道路堤岸桥梁景观改造、绿化景观提升等项目。加快临江大道东延线、车陂路隧道、如意坊隧道及放射线建设。突出抓好琶洲互联网创新集聚区13个重大项目建设，加快国际金融城起步区建设。谋划建设以国际金融城—黄埔临港经济区为核心的第二中央商务区，推进建设白鹅潭中心商务区。

完善大交通综合网络体系。围绕建设国际综合性交通枢纽和国家综合交通枢纽示范城市，推进3个国铁项目、8个城际轨道项目、13个地铁项目、10个高快速路项目、10个国（省）道主干线项目和一批市政道路建设，新扩建一批客运站和货运站，启动建设广汕客专、棠溪站、广州集装箱中心站（大田）、穗莞深城际琶洲支线、广佛江珠城际、地铁18号线、22号线以及一批轨道交通综合枢纽项目，争取广清城际一期、地铁9号线一期、13号线首期、知识城线、4号线南延段、北三环二期、凤凰山隧道项目建成开通。优化调整一批公交线路和站点，建设一批充电桩、换电站、停车场和分布式能源网络，推进出租汽车行业改革和网络约租车、自行车规范管理，有序发展城市慢行系统，建立“枢纽＋社区＋产业”轨道交通建设新模式。

建设南沙城市副中心。完善交通基础设施，构建高快速疏运体系。建设海港、明珠湾起步区、蕉门河中心区、南沙湾4个高品质功能区。发展新能源汽车、高端装备、航运服务、特色金融等现代产业，做大钢铁、有色金属、粮食、塑料等交易中心，打造国家级跨境电商示范基地，吸引一批世界500强和中国500强企业项目落户。深化制度创新，推进在跨境人民币资金业务、证照分离、启运港退税等方面先行先试，推进投资贸易便利化。

（六）优化提升城市功能品质。围绕强化国家重要中心城市功能，树立“精明增长、紧凑城市”理念，加强战略谋划，优化提升干净整洁平安有序城市环境。

规划引领城市发展。着手编制面向2040年的城市总体发展战略规划和交通发展战略规划，有序推进城市总体规划实施，调整完善土地利用总体规划，全面推进“多规合一”。开展全市总体城市设计，注重中心城区优化升级，有序疏解非中心城区功能。制定海绵城市、地下综合管廊、地下管线综合规划和重点功能区规划，完成江心岛控制性详细规划，划定生态控制线。坚持最严格的节约集约用地制度，做好土地收储与精准供给。推进28个功能片区、72个轨道交通站点枢纽综合体和重大基础设施、重大民生工程土地收储。科学合理配置建设用地规模，优先保障重点区域、重大产业项目用地需求。开展批而未用、闲置用地、历史遗留用地的清查和处理工作。

系统推进城市更新。出台贯彻省“三旧”改造和节约集约用地的实施意见，提高土地权利人和市场主体参与的积极性。重点推进87个老旧小区微改造。加强城中村改造和城乡结合部整治。推动一批旧楼宇、产业特色小镇升级改造，整合提升镇村级工业园。建立城市更新土地整备机制，完善历史用地手续，盘活利用国有旧厂房，推进存量低效用地再开发。深化城市更新事权下放，搭建企业和社会资本参与城市更新平台。

持续加强生态环境治理。推进大气污染防治，整治高污染燃料燃烧设施，扩大黄标车限行区域，整改545座加油站油气回收系统，推动船舶废气污染防治，完成60％省级重点监管企业综合整治，实施道路和施工扬尘精细化管理，PM2.5年均浓度进一步下降。推进水环境治理，完成列入国家督办的35条黑臭河涌整治，推进流溪河综合保护治理，石井净水厂投入运营，新改（扩）建9座污水处理厂，力争全市行政村生活污水收集处理全覆盖；建设牛路水库、北江引水、北部水厂饮用水工程，加强饮用水源保护，城市集中式饮用水源地水质持续保证100％达标；完成20项防洪工程。深化垃圾分类投放、分类收集、分类运输和分类处理，持续开展生活垃圾分类示范街镇创建活动，合理布局废旧物资回收网点，引进社会力量参与垃圾治理。加快7个循环经济产业园建设，建成4座资源热力电厂。实施管道燃气三年提升计划。加强土壤面源、环境噪声、重金属、辐射固废、化学品等污染防治。推进国有林场改革。加快绿色生态建设，实施重点林业生态工程和森林城市品质提升工程，完善城市主题花景，新建生态景观林带80公里、森林公园6个、湿地公园3个、碳汇造林4万亩、绿道200公里。

建设新型城镇和美丽乡村。全面推进统筹城乡发展“十三五”规划。深化国家新型城镇化试点，加快农村水利、城镇供水管网、村道建设。建成100个文化（街心）广场，打造“城市微客厅”。实施美丽乡村三年行动计划，创建74条市级、89条区级美丽乡村，精心组织30个特色小镇、17个中心镇和1个镇、15条村的名镇名村建设。加强城乡结合部绿化发展，开展森林小镇创建工作，完善社区公园、镇村公园设施，增加城镇乡村的绿化面积，提高管养水平。

加强城市精细化管理。推进城市管理综合执法、水环境治理、交通行业管理体制改革。建设智慧城市，完善多层级的信息服务网络。创建11条市容环境卫生责任区制度示范街（镇）、30个容貌示范社区，加快招牌示范路建设。整治城市“六乱”和违法建设、违法户外广告、瓶装液化气供应市场、余泥渣土排放，深化交通违法行为和消防安全隐患综合治理，推动市容环境整治向内街小巷延伸。推进基层民主协商共治体系建设，开展社会治理协同创新创建。深入开展来穗人员融合行动，狠抓出租屋专项整治，扩大来穗人员基本公共服务覆盖面。加强流浪乞讨救助服务管理。全面规范街路巷命名，编列建筑物门牌地址。深化网格化服务管理，推进社区服务综合体试点和农村社区试点建设，创新实施“社工＋”。开展村（居）两委换届选举工作。加强人民调解组织建设。打造一批有影响力有社会责任的品牌社会组织。

健全城乡公共安全体系。实行最严格食品药品安全监管，创建国家食品安全城市。健全应急体系和防灾减灾机制。做好春运等重大运输保障。完善自然灾害、极端天气等突发事件预警信息发布体系。深化平安广州建设，健全反恐工作机制，严厉打击违法犯罪活动，开展社会矛盾纠纷排查化解工作，深化禁毒重点整治，推动公共视频联网共享，强化地铁公交的公共安全，打造立体化、信息化社会治安防控体系。推进实施安全生产领域改革，严格落实安全生产责任制，全面开展城市风险点、危险源普查和隐患整治，深化重点行业专项整治，坚决遏制重特大安全事故发生，决不允许以牺牲安全为代价来换取发展。

（七）推进民生社会事业提档升级。坚持为民情怀、民生优先，发挥社会政策托底作用，着力补齐民生短板。

办好十件民生实事。尽管财政收支压力大，但该为群众办的实事一件也不能少。一是加大食品抽查力度；二是加强就业帮扶；三是提高养老服务水平；四是做好母婴室建设和基本公共卫生服务；五是防范打击电信网络诈骗；六是完善公共交通建设和智能化改造；七是加强住房保障；八是下大力治理城市环境；九是落实好文体惠民工作；十是增加基础教育供给和提升学生体质体能。

织密织牢社会保障安全网。推进社保体系创新，推动社会保险扩面，实现省内医疗保险异地就医即时结算，做好失业保险援企稳岗补贴工作。深化养老服务业综合改革，开展国家级医养结合试点、首批长期护理保险制度试点，深化社区居家养老服务改革创新，建立助餐服务网络。抓好医疗救助工作，实施前置救助和支出型救助。健全完善劳动关系矛盾调处机制。深化“羊城慈善为民”行动，创建全国“慈善之城”，大力发展志愿服务事业，使困难群众遇急有助、遇困有帮，让社会充满关爱和温暖。

大力推动社会事业发展。优先发展教育事业，优化教育资源配置，实施中小学校基础教育设施三年提升计划，加强公办和普惠性民办幼儿园建设，推进校园功能微改造，新建一批示范性普通高中，加快职业技术院校迁建工程，推进一流高职院校和高水平大学建设，深化实施校园足球计划。大力普及老年教育，办好一批示范性老年大学。加强基层医疗服务能力建设，推进分级诊疗、基层首诊制度建设，推动“三医”联动和公立医院综合改革，提升妇幼健康和精神卫生服务能力，做好传染病防控和应急救治，加快广州呼吸中心、市胸科医院等项目建设。发展文化事业，加大文化惠民便民力度，做好海上丝绸之路申遗工作，完成史迹点文物本体保护、遗址展示和环境整治工作，推进报业文化中心、广州美术馆、广州文化馆新馆、南粤先贤馆等重大文化基础设施建设，办好中国音乐金钟奖、国际漫画节、广州艺术节、广州文化周、国际艺术博览会、迎春花市等重要文化活动。发展体育事业，推进体育场馆免费或优惠开放，新建一批健身路径，完善社区体育健身设施，办好“市长杯”系列赛、广州马拉松赛、国际龙舟邀请赛，建好全民健身公共服务平台，积极参与国内外竞技体育比赛。做好城市民族工作，推动宗教活动场所合理布局建设。加强“双拥”优抚安置工作，推进军民深度融合发展。深化人民防空与经济社会融合建设。实施残疾预防工作。强化法律服务、法治宣传教育工作，发挥好“一村（社区）一法律顾问”的作用，推进派出所和戒毒场所、监狱、看守所等政法基础设施建设。依法维护海外侨胞和港澳台同胞的合法权益，充分发挥他们的独特优势和重要作用。扎实做好国防动员、人口计生、海防、打私、打假、统计、应急、气象、地震、参事、文史、档案、保密、修志等工作。

（八）加强政府自身建设。坚决执行党中央制定的方针政策，坚决落实党中央确定的改革方案，牢记宗旨，勤勉尽责，依法行政，公正用权，切实做到对人民负责、受人民监督、让人民满意。

加强法治政府建设。严格遵守宪法和法律，依法接受市人大及其常委会的监督，自觉接受市政协的民主监督，主动接受社会和舆论监督。依法分类处理信访诉求。深入推进重大民生决策公众咨询监督、网络问政、新闻发布会等制度，加强重大行政决策、规范性文件合法性审查，依法审理行政复议案件，全面落实政府法律顾问制度。

打造服务型政府。推进政府决策、执行、管理、服务、结果公开，规范受理、依法答复和公开政府信息申请，分批出台公共服务事项目录和办事指南。推行“互联网＋政务服务”改革，推进审批服务事项一窗受理、网上办理、全城通办，促进实体政务大厅与网上办事大厅融合，提升12345热线综合服务能力和服务水平，让数据多跑路，让群众少跑腿。

建设廉洁高效政府。严明政治纪律和政治规矩，严格落实中央八项规定精神，驰而不息纠正“四风”，大力整治“为官不为”问题。加强农村、社区、基层服务、扶贫等领域监督执纪问责。强化行政监察，推进审计机关人财物管理改革和审计全覆盖。深化国企巡察和廉洁南沙自贸试验区建设，开展政府投资项目廉洁评估，建立“亲”“清”新型政商关系，打造廉洁政府。强化责任担当，发扬钉钉子精神，大兴调查研究之风，狠抓工作落实。完善政绩考核评估体系，充分调动干部职工工作积极性、主动性、创造性，让干部职工愿干事、敢干事、能干成事。

各位代表，实现宏伟的目标，需要我们攻坚克难，干字当头，我们要以实干走向未来，也只有实干才能赢得未来。让我们紧密团结在以习近平同志为核心的党中央周围，牢固树立“四个意识”特别是核心意识、看齐意识，在市委的坚强领导下，坚定信心，奋发有为，不忘初心，继续前进，为建设枢纽型网络城市、推进国家重要中心城市建设全面上水平而努力奋斗！
